# Effects of indacaterol on the LPS-evoked changes in fluid secretion rate and pH in swine tracheal membrane

**DOI:** 10.1007/s00424-021-02560-z

**Published:** 2021-05-24

**Authors:** Hidemi Aritake, Tsutomu Tamada, Koji Murakami, Shunichi Gamo, Masayuki Nara, Itsuro Kazama, Masakazu Ichinose, Hisatoshi Sugiura

**Affiliations:** 1 Department of Respiratory Medicine, Tohoku University Graduate School of Medicine, 1-1 Seiryo-machi, Aoba-ku, Sendai, 980-8574 Japan; 2 National Hospital Organization Akita National Hospital, Yurihonjo, Japan; 3 Miyagi University School of Nursing Graduate School of Nursing, Kurokawa-gun, Japan; 4 Osaki Citizen Hospital, Osaki, Japan

**Keywords:** LABA, Bicarbonate, CFTR, Calu-3, Submucosal gland

## Abstract

An acquired dysregulation of airway secretion is likely involved in the pathophysiology of chronic bronchitis and chronic obstructive pulmonary disease (COPD). Nowadays, it is widely known that several kinds of long-acting bronchodilators reduce the frequency of COPD exacerbations. However, limited data are available concerning the complementary additive effects on airflow obstruction. Using an optical method and a selective pH indicator, we succeeded in evaluating the gland secretion rate and the pH in swine tracheal membrane. A physiologically relevant concentration of acetylcholine (ACh) 100 nM induced a gradual increase in the amount of gland secretion. Lipopolysaccharides (LPS) accelerated the ACh-induced secretory responses up to around threefold and lowered the pH level significantly. Long-acting β_2_-agonists (LABAs) including indacaterol (IND), formoterol, and salmeterol restored the LPS-induced changes in both the hypersecretion and acidification. The subsequent addition of the long-acting muscarine antagonist, glycopyrronium, further increased the pH values. Two different inhibitors for cystic fibrosis transmembrane conductance regulator (CFTR), NPPB and CFTR_inh172_, abolished the IND-mediated pH normalization in the presence of both ACh and ACh + LPS. Both immunofluorescence staining and western blotting analysis revealed that LPS downregulated the abundant expression of CFTR protein. However, IND did not restore the LPS-induced decrease in CFTR expression on Calu-3 cells. These findings suggest that the activation of cAMP-dependent HCO_3_^−^ secretion through CFTR would be partly involved in the IND-mediated pH normalization in gland secretion and may be suitable for the maintenance of airway defense against exacerbating factors including LPS.

**Supplementary Information:**

The online version contains supplementary material available at 10.1007/s00424-021-02560-z.

## Introduction

Airway surface liquid (ASL) contains mucin, antimicrobial proteins, electrolytes (and therefore water), and immunoglobulins, all of which contribute to the maintenance of the airway defense [31]. Because human airway epithelium is likely to be primarily absorptive [4, 51], a major fraction of the airway fluid is derived from submucosal glands (SMGs) [13, 33]. Concerning the anion secretion from tracheal SMGs, two major pathways have been identified. One is characterized by the cystic fibrosis transmembrane conductance regulator (CFTR) channel, which is mainly activated by cAMP and conducts both Cl^−^ and HCO_3_^−^; the other is characterized by the Ca^2+^-activated chloride channel (CaCC), which conducts only Cl^−^. These channels widely exist in SMGs as well as the airway surface epithelium [23, 29]. An in vivo study has demonstrated that cholinergic agents are much more potent stimulators of gland secretion than adrenergic agonists, as ascertained by the hillock formations from a powdered tantalum layer coating the airway surface [27]. In contrast, CFTR channels are known to secrete HCO_3_^−^ in response to a cAMP-mediated agonist, but can also be stimulated to secrete Cl^−^ by the activation of the basolateral membrane Ca^2+^-activated K^+^ channel [5, 10, 18, 45]. Recent studies have demonstrated that the abnormal acidification in the ASL pH is related to a defect in cAMP-dependent HCO_3_^−^ secretion through CFTR and initiates host defense abnormalities in cystic fibrosis [16, 39, 40]. These findings suggest that HCO_3_^−^ secretion may enhance airway host defenses by increasing the ASL pH, decreasing the ASL viscosity, increasing the activity of antimicrobial factors, and counteracting local environmental acidification by some bacteria [27].

Lipopolysaccharides (LPS) exist ubiquitously in the outer membrane of most Gram-negative bacteria including *Pseudomonas aeruginosa* (*P. aeruginosa*). It is known that *P. aeruginosa* often colonizes the airways of patients with chronic obstructive pulmonary disease (COPD) and sometimes causes repeated respiratory infections, resulting in the development of COPD exacerbations [42]. *Pseudomonas aeruginosa* and its derivatives reduce CFTR-mediated transepithelial anion secretion across polarized human airway epithelium [22, 24, 38, 43, 48]. Additionally, cigarette smoke itself decreases the expression of CFTR protein and function in vitro [8]. Smokers with and without COPD have lower airway CFTR activity compared to healthy nonsmokers, and this reduced activity correlates with the disease phenotype [11]. These findings suggest that an acquired CFTR dysfunction is likely to be involved in the pathophysiology of chronic bronchitis or COPD, and even in an increased risk of COPD exacerbations [11].

Nowadays, long-acting bronchodilators (LABDs) such as long-acting muscarinic antagonist (LAMA) or long-acting β_2_ agonist (LABA) alone and in combination enable great reductions in the frequency of COPD exacerbations as well as significant improvements in lung function [7, 28, 49, 50]. Although direct anti-inflammatory effects have not been demonstrated yet in patients with COPD, preclinical studies suggest complementary additive effects of LAMA on airflow obstruction [3]. Patients treated with tiotropium bromide, one of the most popular LAMAs, have shown a subjective decrease in sputum production [32], a significant improvement in mucociliary clearance [44] and several effects on inflammatory cells in in vitro studies [1, 2, 21, 30]. In contrast, concerning several LABAs such as indacaterol maleate (IND), formoterol fumarate (FOR), and salbutamol sulfate (SAL), limited data are available concerning the complementary additive effects on airflow obstruction.

In the present study, we investigated the effects of LPS on the gland secretion rate and the pH value in swine tracheal membranes and the ameliorating effects of LABAs on the LPS-evoked abnormal changes.

## Materials and methods

All methods are presented in greater detail in the online supplement.

### Preparation of swine tracheal tissues

Fresh swine tracheas were obtained at a local slaughterhouse, cut into rings 3–4 cm and fixed by pins with the apical wall side up. Details are described in our previous reports [14, 25, 26, 46, 47].

### Analysis of the amount of gland secretion in swine tracheal membrane

Using an optical method reported by Joo NS and colleagues [20], we succeeded in a real-time evaluation of the amount of physiologic airway secretion from swine tracheal SMGs.

### Preparation of cells

For the patch-clamp experiments, fresh SMG cells were isolated from swine tracheas and dispersed enzymatically into single or clustered acinar cells. Details are described in our previous reports [14, 25, 26, 46, 47]. Calu-3 cells purchased from the American Type Culture Collection (ATCC, Rockville, MD) were grown on Snapwell filters as previously described [10] and studied in a horizontal chamber.

### Electrophysiology

Ionic currents were measured according to a standard whole-cell mode patch-clamp technique using a patch-clamp amplifier (EPC9; HEKA Electronic, Lambrecht/Pfalz, Germany). Using proper channel inhibitors and ion substitution experiments, we have reported that the ACh-induced outward current (*I*_o_) and inward current (*I*_i_) were carried mainly by K^+^ and Cl^−^, respectively, which were dependent on [Ca^2+^]_i_ [19, 36, 37]. Details are described in our previous reports [14, 25, 26, 46, 47].

### Quantification procedure

The procedure to evaluate the ionic responses using the area circumscribed with the current trace (*I*_o_ or *I*_i_) and baseline for 20 s (= area under curve_20_) was also applied in our previous reports [14, 25, 26, 46, 47].

### Analysis of the ASL pH in swine tracheal membrane

Individual SMG secretions were collected from swine tracheal membrane overlaid with a mineral oil. Using pH indicator, SNARF-1 (Thermo Fisher Scientific, Waltham, MA, USA) [15] and Flexstation 3 microplate reader (Molecular Devices, Sunnyvale, CA, USA), the ASL pH was evaluated automatically.

### Analysis of apical surface liquid pH on Calu-3 cells

Calu-3 cells with double barrel voltage and pH electrodes were used in a horizontal chamber that enabled estimation of the pH values just above the apical membrane of the Calu-3 cells. Details are described in previous reports [10, 18, 45].

### Immunofluorescence staining

Immunofluorescence staining was performed to detect CFTR as described previously [14, 25, 26].

### Western blotting

Calu-3 cells grown on 6-well culture plates were incubated in a medium containing LPS (0, 10, and 100 μg/ml) with or without IND (1 µM) for 3 h. Details are described in our previous reports [14, 25, 26, 46].

### Statistical analysis

All analyses were performed using JMP Pro 14 (SAS Institute Inc., Cary, NC, USA). The data are expressed as means ± standard errors (SE); *n* is the number of experiments in different animals. Electrophysiological experiments were analyzed by the Wilcoxon signed rank test. The amount of airway secretion and the ASL pH were analyzed by the Wilcoxon signed rank test and matched paired *t* test, respectively. Statistical significance was accepted at *p* < 0.05, indicated by asterisks or other symbols in all figures.

### Reagents

IND was provided by Novartis Pharma AG (Basel, Switzerland). See the online supplement for more details.

## Results

### Visualization of ACh-induced gland secretion in swine tracheal mucosa

**Fig. 1:**
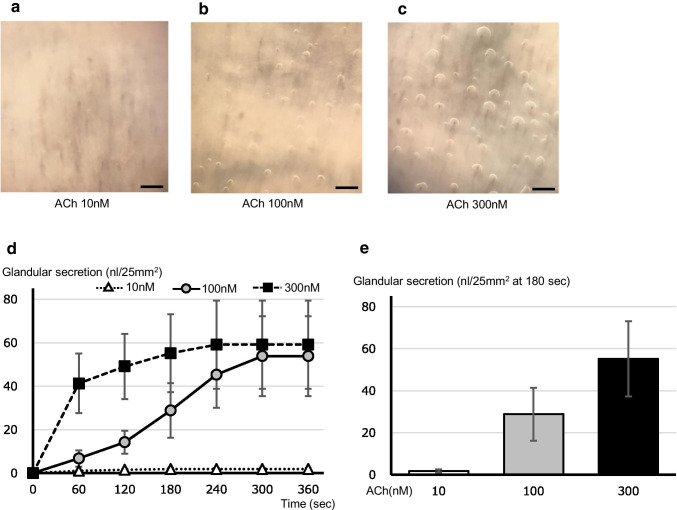
Representative appearances showing the hillock formations of gland secretion in swine tracheal mucosa. **a**-**c** Through a mineral oil layer coating the airway surface, the hillock formations of swine tracheal gland secretion at 180 s after the stimulation by ACh (10, 100, and 300 nM, respectively) were visualized by means of stereoscopic microscope. **d** Time courses of the changes in the total amount of gland secretion per 25 mm^2^ stimulated by several doses of ACh. **e** Comparisons of the total amount of gland secretion per 25 mm^2^ at 180 s after stimulation by several doses of ACh. Scale bars: 500 µm

Representative appearances of gland secretion in swine tracheal mucosa at 180 s after the stimulation by several doses of ACh are shown in Fig. 1. Although ACh (10 nM) did not generate any secretory responses (Fig. 1a), the apparent hillock formations of the mineral oil layer coating the airway surface were observed under a stereoscopic microscope after the stimulation by ACh (100 and 300 nM) (Fig. 1b-c). A summary of changes in the amount of secretions per 25mm^2^ was plotted in Fig. 1d. ACh (300 nM) rapidly increased the amount of gland secretion, and caused near maximum responses within as short as 60 s. In contrast, ACh (100 nM) increased the secretion gradually, and the degree of the secretory response at 180 s was almost half of that by ACh (300 nM) (28.8 ± 12.6 for ACh 100 nM and 53.8 ± 18.4 nl/25mm^2^ at 180 s for ACh 300 nM, respectively, Fig. 1e). From these data, we considered that the stimulation by 100 nM of ACh was suitable for the observation of physiologically relevant gland secretion in our experimental setting.

### Effects of LPS on the amount of ACh-induced gland secretion

**Fig. 2:**
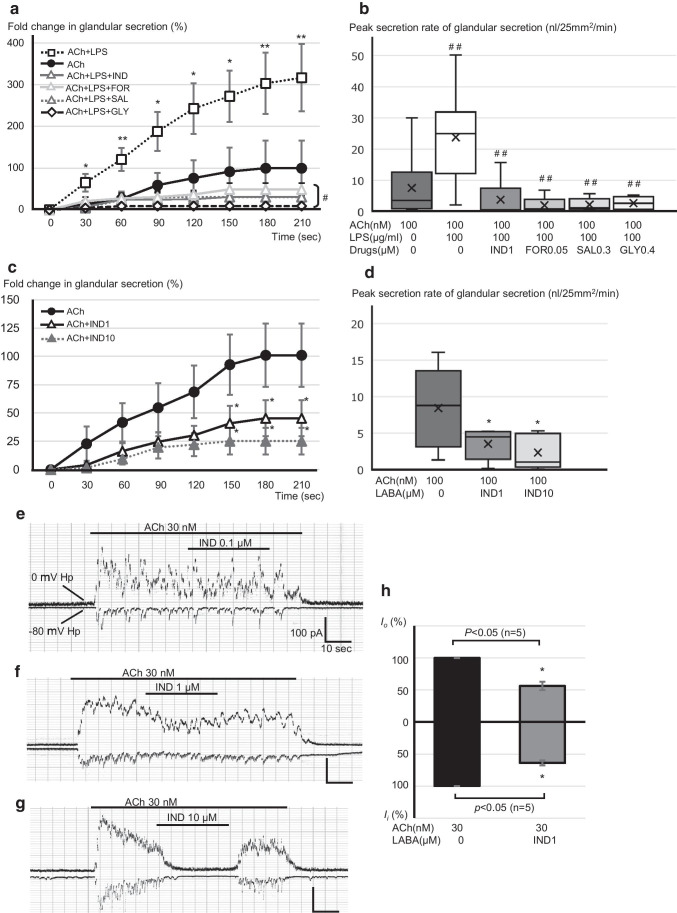
Effects of LPS and/or LABAs on the amount of ACh-induced gland secretion in swine trachea. **a** Time courses of the changes in the amount of gland secretion induced by the indicated stimulations, that is, ACh 100 nM alone (*n* = 9); black solid line/black circle, ACh + LPS (100 µg/ml) (*n* = 9); black dotted line/white square, ACh/LPS + IND (1 µM) (*n* = 7); dark gray solid line/white triangle, ACh/LPS + FOR (0.05 µM) (*n* = 5); dark gray dotted line/white triangle, ACh/LPS + SAL (0.3 µM) (*n* = 5); light gray solid line/white triangle, and ACh/LPS + GLY (0.4 µM) (*n* = 5); black dashed line/white diamond. These responses were compared by estimating the mean values of the ACh responses at 180 s as 100% and data are shown as mean ± SE. **b**, **d** Comparisons of the peak secretion rate induced by the indicated stimulations. In the box plots, the boundary of the box closest to zero indicates the 25th percentile, a black line within the box marks the median, a cross mark in each box marks the mean, and the boundary of the box farthest from zero indicates the 75th percentile. Whiskers above and below the box indicate the 10th and 90th percentiles. **c** Time courses of the changes in the amount of gland secretion induced by the indicated stimulations, that is, ACh 100 nM alone (*n* = 5), black solid line/white circle; ACh + IND (1 µM) (*n* = 5), dark gray solid line/white triangle; and ACh + IND (10 µM) (*n* = 5), dark gray dotted line/white triangle. **e**–**g** Representative original recordings delineating the effects of several doses of IND (0.1, 1, and 10 µM, respectively) on ACh (30 nM)-evoked ionic currents by means of whole-cell patch-clamp recording. **h** Summary of the effects of IND (1 µM) on ACh 30 nM-evoked *I*_o_ and *I*_i_ (*n* = 5). Five sets of reliable patch-clamp data are from 5 different swine tracheas. Hp: holding potential; *p < 0.05 vs. ACh, **p < 0.01 vs. ACh, #*p* < 0.05 vs. ACh + LPS. ##*p* < 0.01 vs. ACh + LPS

We next investigated the effects of LPS on the amount of airway secretion under the stimulation by ACh (100 nM). Compared with the cases of ACh alone (solid line/black circle), LPS (100 µg/ml) in combination with ACh significantly increased the amount of gland secretion in a time-dependent manner (Fig. 2a, dashed line/white box). At 180 s after the stimulation by ACh/LPS, the amount of gland secretion increased to threefold (303.2 ± 73.6%) when compared by estimating the mean values of the ACh responses at 180 s as 100%. As shown in Fig. 2b, the peak secretion rates after the stimulation by ACh/LPS increased to threefold compared with those by ACh alone (7.5 ± 3.3 for ACh, and 23.8 ± 4.8 nl/25mm^2^/min for ACh/LPS, white box). These findings suggest that LPS causes a significant potentiation in the physiologically relevant gland secretion in swine tracheal mucosa.

### Effects of LABAs on the amount of ACh-induced gland secretion

We further investigated whether clinically used LABDs attenuate the LPS-induced potentiation in the rate of gland secretion. As shown in Fig. 2a (triangles), each of the three LABAs (IND, FOR, and SAL) caused a remarkable attenuation in the LPS-induced hypersecretion. Glycopyrronium (GLY, 0.4 µM), one of the popular LAMAs, also caused the same attenuation (Fig. 2a, diamond). The effects of these three LABAs or GLY on the peak secretion rates after the stimulation by ACh/LPS are summarized in Fig. 2b. All LABAs or GLY attenuated the LPS-induced acceleration in the peak secretion rates to less than half when compared with the mean values by ACh alone (3.8 ± 2.2 for ACh/LPS/IND, 1.9 ± 1.2 for ACh/LPS/FOR, 2.2 ± 0.9 for ACh/LPS/SAL, and 2.7 ± 1.1 nl/25mm^2^/min for ACh/LPS/GLY, Fig. 2b, gray boxes). Notably, even in the absence of LPS, 1 and 10 µM of IND showed a significant attenuation in the amount of ACh-induced gland secretion at 180 s to less than half (45.4 ± 15.6% for ACh/IND1 and 25.1 ± 11.7% for ACh/IND10, respectively, Fig. 2c) when compared by estimating the mean values of the ACh response at 180 s as 100%. Likewise, 1 and 10 µM of IND showed a significant attenuation in the peak secretion rates to less than half (8.4 ± 2.5 for ACh, 3.5 ± 1.0 for ACh/IND1, and 2.3 ± 1.1 nl/25mm^2^/min for ACh/IND10, Fig. 2d). To further determine the mechanisms underlying the IND-mediated attenuation in the gland secretion, we investigated the effects of IND on the ACh-evoked ionic currents using patch-clamp experiments. As shown in Fig. 2e-g, IND suppressed these ionic currents in a dose-dependent manner. Data summarizing the effects of IND (1 µM) on the ACh (30 nM)-evoked ionic currents show that both *I*_o_ and *I*_i_ decreased to nearly two-thirds (*I*_o_: 139.5 ± 20.0 pQ/s for ACh vs. 76.8 ± 9.9 pQ/s for ACh/IND, and *I*_i_: 41.5 ± 5.5 pQ/s for ACh, and 26.5 ± 3.6 pQ/s for ACh/IND, *p* < 0.05, *n* = 7, Fig. 2h). These findings suggest that each of the three LABAs has the potency to cause a remarkable attenuation in the LPS-induced hypersecretion, probably by inhibiting ionic currents through CaCC.

### Effects of LABAs on the pH of ACh-induced gland secretion

**Fig. 3:**
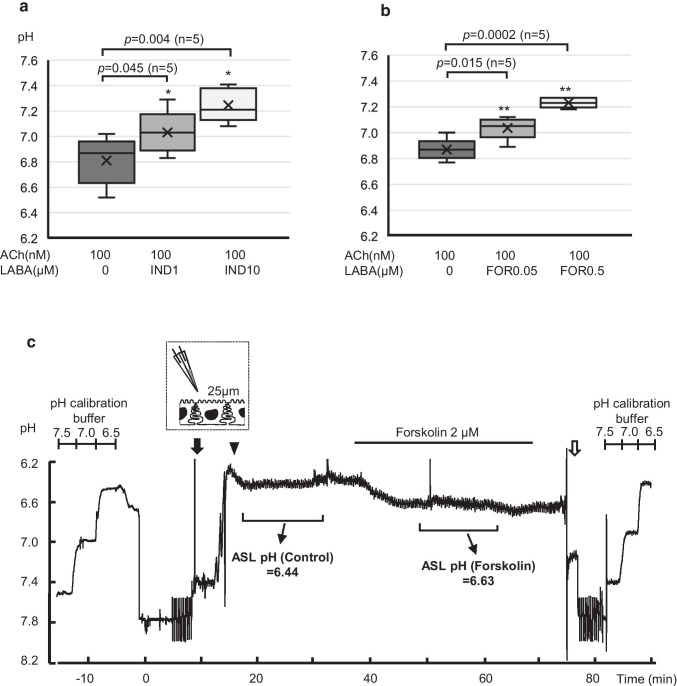
Effects of cAMP-increasing agents on pH values in ACh-induced gland secretion in swine tracheal mucosa. **a** Summary of the pH values of gland secretion induced by ACh in combination with IND (0, 1, and 10 µM). For the explanation of the box plots, see Fig. 2 legend. **b** Summary of the pH values of gland secretion induced by ACh in combination with FOR (0, 0.05, and 0.5 µM). *p < 0.05 vs. ACh, **p < 0.01 vs. ACh. **c** Representative original recording delineating the effect of forskolin on the ASL pH of Calu-3 cells. After calibration of the pH electrode in a series of buffer solutions (pH = 7.5, 7.0, and 6.5), a double barrel voltage and pH microelectrode was inserted carefully into the apical solution with low buffering capacity (black arrow). As the microelectrode went down toward the apical membrane, the pH values became more acidic. When the tip of the pH microelectrode reached to 25 μm above the apical surface (arrow head), the electrode was held and the pH values were analyzed continuously. After the pH values became stable, forskolin (2 µM) was added to the basolateral solution during the indicated period. After the experiment, the pH microelectrode was pulled out of the chamber (white arrow) and calibrated again with buffer solution

Using a pH selective indicator, SNARF-1, we analyzed the pH values of gland secretion in swine tracheal mucosa. The mean pH values under the stimulation by ACh alone were around 6.81 ± 0.08, and the subsequent addition of 1 or 10 µM of IND showed significant changes in pH to 7.03 ± 0.08 (ΔpH = 0.22, *p* = 0.045, *n* = 5) or 7.25 ± 0.06 (ΔpH = 0.44, *p* = 0.004, *n* = 5), respectively (Fig. 3a). Likewise, the subsequent addition of 0.05 or 0.5 µM of FOR showed significant changes in pH from 6.87 ± 0.03 to 7.04 ± 0.04 (ΔpH = 0.17, *p* = 0.015, *n* = 5) or 7.23 ± 0.02 (ΔpH = 0.36, *p* = 0.0002, *n* = 5), respectively (Fig. 3b). To determine the involvement of intracellular cAMP-dependent mechanisms, we further investigated the effects of forskolin, a cAMP-increasing agent, on the ASL pH in Calu-3 cells by means of microelectrode experiments. A representative tracing indicating the pH values measured by a pH-sensitive microelectrode held at 25 μm above the apical surface of the Calu-3 cells is shown in Fig. 3c. The addition of forskolin (2 µM) showed apparent alkaline changes in the mean pH value from 6.44 to 6.63 (ΔpH = 0.19). These findings suggest that clinically used LABAs have the potency to raise the ASL pH toward alkaline in a dose-dependent manner via intracellular cAMP-dependent mechanisms.

### Effects of LPS and LABAs on the pH of ACh-induced gland secretion

**Fig. 4:**
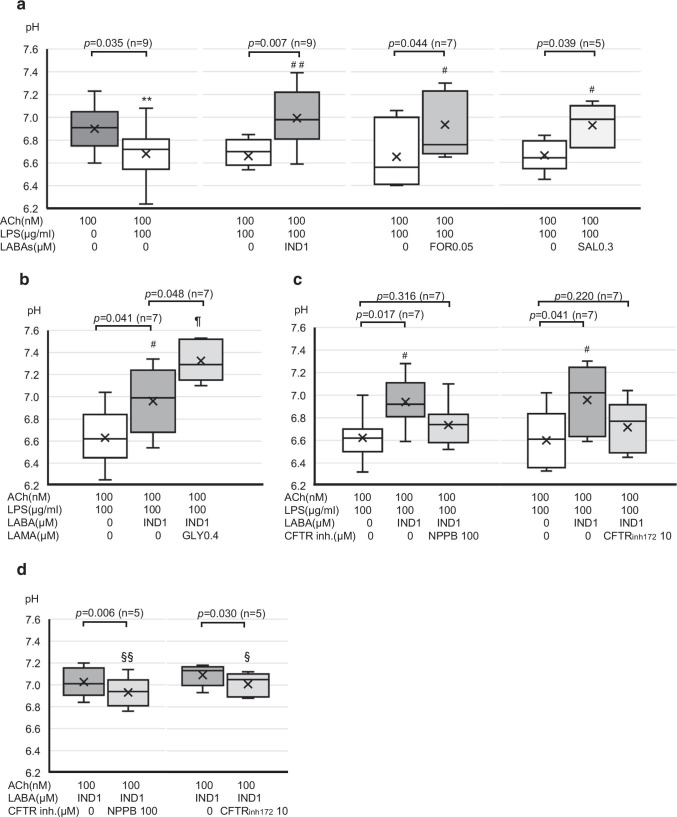
Effects of LPS, LABAs, and CFTR inhibitors on the pH of ACh-induced gland secretion in swine tracheal mucosa. **a** Effects of LPS (100 µg/ml) on the pH of ACh-induced gland secretion (left panel), and those of LABAs (IND 1 µM, FOR 0.05 µM and SAL 0.3 µM) on the pH of both ACh and LPS-induced gland secretion (right three panels). For the explanation of the box plots, see Fig. 2 legend. **b** Effects of GLY (0.4 µM) in combination with IND (1 µM) on the pH of gland secretion under the stimulation by both ACh and LPS. **c** Loss of effects of IND on the pH of gland secretion induced by both ACh and LPS in the presence of CFTR inhibitors, NPPB (100 µM) and CFTR_inh172_ (10 µM). **d** Effects of CFTR inhibitors alone on the pH of both ACh and IND-induced gland secretion. **p < 0.01 vs. ACh, #*p* < 0.05 vs. ACh + LPS, ##*p* < 0.01 vs. ACh + LPS, **¶p** < 0.01 vs. ACh + LPS + IND, §*p* < 0.05 vs. ACh + IND, §§*p* < 0.01 vs. ACh + IND

We next investigated the effects of LPS on the pH of ACh-induced gland secretion. As shown in Fig. 4a (left panel), the subsequent addition of LPS (100 µg/ml) showed significant acidic changes in pH (6.90 ± 0.07 for ACh alone, and 6.68 ± 0.08 for ACh/LPS, ΔpH =  − 0.22, *p* = 0.035, *n* = 9). Notably, even in the presence of LPS, the subsequent addition of IND showed significant alkaline changes in the pH (6.66 ± 0.08 for ACh/LPS, and 6.99 ± 0.09 for ACh/LPS/IND, ΔpH = 0.33, *p* = 0.007, *n* = 9). Likewise, the addition of FOR changed the pH values from 6.65 ± 0.10 for ACh/LPS to 6.94 ± 0.11 for ACh/LPS/FOR (ΔpH = 0.29, *p* = 0.044, *n* = 7), and SAL also changed them from 6.66 ± 0.06 for ACh/LPS to 6.93 ± 0.08 for ACh/LPS/SAL (ΔpH = 0.27, *p* = 0.039, *n* = 5) (Fig. 4a, right three panels). Previous reports [34, 47] described a negative cross-talk between muscarinic and beta-adrenergic receptors in the intracellular signaling of airway gland secretion. The addition of GLY in combination with IND induced further significant alkaline changes in pH (6.96 ± 0.14 for ACh/LPS/IND, and 7.32 ± 0.07 for ACh/LPS/IND/GLY, ΔpH = 0.36, *p* = 0.006, *n* = 7) (Fig. 4b). These findings suggest that LPS causes a remarkable impact in the pH values of ACh-induced gland secretion and that all three LABAs and GLY in combination with IND have the potency to restore the LPS-induced acidification.

### CFTR inhibition attenuates the IND-mediated improvement of the LPS-evoked acidic changes in pH of the ACh-induced gland secretion

As the ASL pH is greatly dependent on the cAMP-dependent HCO_3_^−^ secretion thorough CFTR [16, 39, 40], we next investigated the effects of CFTR channel inhibitors on the LABA-mediated improvement of the LPS-induced acidification in pH. When tracheal membranes were preincubated with NPPB (100 µM), a specific CFTR inhibitor, IND did not show significant changes in pH (6.62 ± 0.08 for ACh/LPS, and 6.74 ± 0.07 for ACh/LPS/IND/NPPB, *p* = 0.316, *n* = 7), while IND did in the absence of NPPB (6.94 ± 0.08 for ACh/LPS/IND, *p* = 0.017, *n* = 7) (Fig. 4c, left panel). Likewise, in the presence of CFTR_inh172_ (10 µM), another specific CFTR channel blocker, IND did not show significant changes in pH (6.67 ± 0.10 for ACh/LPS, and 6.77 ± 0.08 for ACh/LPS/IND/CFTR_inh172_, *p* = 0.220, *n* = 7), while IND did in the absence of CFTR_inh172_ (6.99 ± 0.10 for ACh/LPS/IND, *p* = 0.041, *n* = 7) (Fig. 4c, right panel). To confirm the involvement of CFTR in the IND-mediated pH normalization, we investigated the effects of these two CFTR inhibitors on the pH of the gland secretion induced by ACh/IND. In the presence of NPPB or CFTR_inh172_, the pH values showed significant changes from 7.03 ± 0.06 for ACh/IND to 6.93 ± 0.06 for ACh/IND/NPPB (*p* = 0.006, *n* = 5) or from 7.09 ± 0.04 for ACh/IND to 7.01 ± 0.05 for ACh/IND/ CFTR_inh172_ (*p* = 0.030, *n* = 5), respectively (Fig. 4d). But these restorations were incomplete when compared to the pH values for ACh alone (6.81–6.87, see Fig. 3). These findings suggest that the activation of CFTR is likely to be partly involved in the IND-mediated improvement of LPS-induced acidification in pH and that LABAs may have the potency to improve cAMP-dependent HCO_3_^−^ secretion through CFTR in combination with other CFTR-independent pH regulatory mechanisms.

### Mechanisms underlying the CFTR-dependent improvement of LPS-evoked acidic changes in pH

**Fig. 5:**
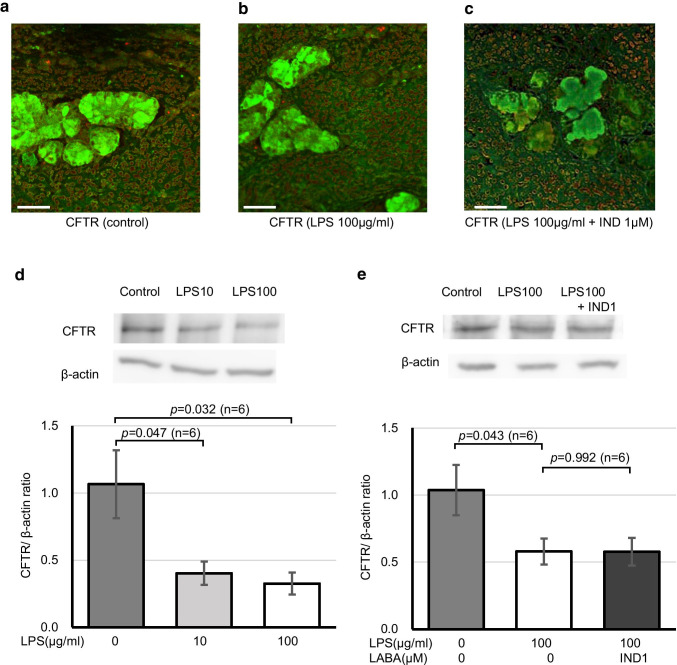
Expression of CFTR protein in swine tracheal SMGs and Calu-3 cells. **a**-**c** Immunofluorescence images showing overlap of CFTR (green) with plasma membrane (red). Swine tracheal tissues were preincubated without (**a**), with LPS (100 µg/ml) (**b**), and with LPS (100 µg/ml) + IND (1 µM) (**c**) for 10 min. Scale bars: 100 µm. **d**, **e** Western blotting analysis of Calu-3 cells using CFTR protein. Representative images of CFTR bands (168kD) and β-actin (42kD) are shown in the upper panels. Summary of the ratios of CFTR to β-actin protein by means of western blotting analysis are shown in lower panels (*n* = 6 for each graph)

An acquired dysfunction of CFTR is theoretically caused by two mechanisms. One is an inhibition of CFTR channel activity, and the other is a downregulation of CFTR protein expression on the plasma membrane. Concerning the latter, the immunofluorescence double staining experiments showed abundant localization of CFTR protein in the swine tracheal SMGs (Fig. 5a, green for CFTR and red for plasma membrane). When the tracheal tissues were preincubated in a medium containing LPS with or without IND for 10 min at room temperature, the overlap signaling showed a tendency to decrease in both cases (Fig. 5b, c). Western blot quantification revealed that LPS caused a significant decrease in the ratio of CFTR/β-actin protein on Calu-3 cells (1.06 ± 0.25 for control, 0.40 ± 0.09 for LPS 10 µg/ml, *p* = 0.047, *n* = 6, and 0.33 ± 0.08 for LPS 100 µg/ml, *p* = 0.032, *n* = 6) (Fig. 5d). However, IND (1 µM) did not improve the LPS-induced decrease in the expression of CFTR protein (Fig. 5e). These findings suggest that, while LPS has the potency to downregulate the abundant expression of CFTR on the plasma membrane, the IND-mediated improvement of LPS-evoked acidification in pH may not be caused by the upregulation of CFTR protein expression, and it is likely due to the upregulation of cAMP-dependent HCO_3_^−^ secretion through CFTR in combination with other CFTR-independent pH regulatory mechanisms.

## Discussion

In the present study, we demonstrated that, in the gland secretion from fresh swine tracheal membrane, LPS caused a significant increase in the ACh-induced secretion rate by threefold (Fig. 2a) and an acidic change in pH of 0.2 (Fig. 4a). We further demonstrated that IND and other clinically used LABAs restored both the LPS-induced hypersecretion (Fig. 2a-b) and acidification (Fig. 4a). Although LPS downregulated the expression of CFTR on the cells, IND improved the LPS-evoked acidification in pH without restoration of the expression of CFTR (Fig. 5e). To our knowledge, this is the first report demonstrating a positive impact of LABAs on LPS-induced pathological changes in the gland secretion rate and the pH in swine tracheal membrane.

**Fig. 6:**
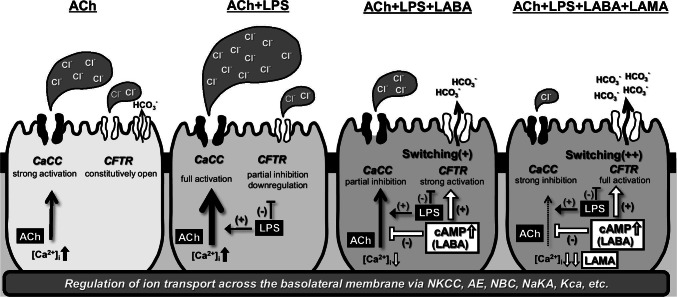
A graphical summary of the findings in this study. Previous reports [34, 47] suggest that a negative cross-talk between muscarinic and beta-adrenergic receptors exists in the intracellular signaling of airway gland secretion. Therefore, cAMP-increasing agents such as IND or other LABAs can greatly attenuate the ACh-induced [Ca^2+^]_i_-dependent electric responses. Other previous reports [5, 45] suggest that, Calu-3 cells secrete HCO_3_^−^ through CFTR in response to a cAMP-mediated agonist but can be stimulated to secrete Cl^−^ with an ACh-like stimulation. If this “switching” mechanism is conserved in the regulation of tracheal gland secretion, it is then likely to explain our findings. When cells are stimulated by ACh or ACh + LPS, tracheal glands secret HCO_3_^−^ free, Cl^−^ rich, and large amount of fluid, which tend to be relatively acidic in pH. The addition of IND or other LABAs is likely to induce the “switching” mechanism from Cl^−^ to HCO_3_^−^ secretion through CFTR, accelerate singular HCO_3_^−^ secretion, and induce remarkable alkaline changes in pH, although it will in turn simultaneously cause a significant decrease in the amount of fluid. Anti-cholinergic agent such as GLY will further induce the “switching” mechanism from Cl^−^ to HCO_3_^−^ secretion through CFTR and accelerate the IND-mediated pH normalization in gland secretion. NKCC: Na^+^/K^+^/2Cl^−^ cotransporter; AE: HCO_3_^−^/Cl^−^ anion exchanger; NBC: Na^+^/3HCO_3_^−^ cotransporter; NaKA: Na^+^-K^+^ ATPase; Kca: Ca^2+^-activated K^+^ channel

There are two major pathways of anion secretion from tracheal SMGs, CFTR and CaCC [23, 29]. It is widely known that the Ca^2+^-dependent Cl^−^ secretion through CaCC contributes to the volume of the gland secretion [23, 27, 29], while the cAMP-dependent HCO_3_^−^ secretion thorough CFTR contributes to the maintenance of the ASL pH [16, 39, 40]. Concerning the effects of LPS on the physiological function of CaCC, we have reported that, using patch-clamp experiments, LPS showed a significant potentiation in ACh-induced, Ca^2+^-dependent ionic currents from swine tracheal SMG cells via a toll-like receptor 4 [25]. Likewise, Buyck and colleagues have shown that LPS from *P. aeruginosa* stimulated Ca^2+^ signaling and Cl^−^ secretion in a human bronchial epithelial cell line [6]. In contrast, we have reported that isoproterenol or a cocktail of 3-isobutyl-1-methylxanthine (IBMX) and 8-(4-chlorophenylthio)-cAMP (cpt-cAMP) caused a nearly complete inhibition of the ACh-evoked ionic currents in cat tracheal SMG cells [47]. Cross-talk between muscarinic cholinoceptors and β_2_-adrenoceptor is likely to be present and cause a negative impact on the muscarinic stimulation of airway smooth muscle cells [34]. In line with these findings, the present study revealed that IND attenuated the ACh-induced, Ca^2+^-dependent ionic currents in swine tracheal SMG cells in a dose-dependent manner (Fig. 2e-g) and that three LABAs attenuated the LPS-induced hypersecretion (Fig. 2a-b). As described in previous reports [34, 47], we think that a negative cross-talk between muscarinic and beta-adrenergic receptors is likely to exist in the intracellular signaling of airway gland secretion. Concerning the changes in pH of gland secretion, there are several pH regulatory mechanisms such as cAMP-dependent HCO_3_^−^ secretion through CFTR, HCO_3_^−^/Cl^−^ anion exchanger, electrogenic Na^+^/HCO_3_^−^ cotransporter, and some other electroneutral and/or bicarbonate-independent mechanisms [17]. In the present study, we demonstrated that LPS caused an acidic change in the pH (Fig. 4a, left panel), IND and other LABAs restored the LPS-induced acidification (Fig. 4a, right three panels), forskolin caused apparent alkaline changes in the ASL pH on Calu-3 cells (Fig. 3c), and CFTR inhibitors abolished the IND-mediated improvement in acidified pH values with and without LPS (Fig. 4c, d), but these restorations were incomplete when compared to the pH values for ACh alone. There are two possibilities regarding the CFTR-dependent acidification in pH: One is a downregulation of CFTR expression on the plasma membrane, and the other is an inhibition of CFTR channel activity. In the present study, we showed that CFTR protein was abundantly expressed on swine tracheal SMGs and that the LPS decreased the expression of CFTR protein in both immunofluorescent staining (Fig. 5a, c) and western blotting (Fig. 5d, e). However, IND did not restore the expression of CFTR protein (Fig. 5e). These findings suggest that the IND-induced pH normalization is determined by both CFTR-dependent and CFTR-independent mechanisms. It seems that, in addition to cAMP-dependent HCO_3_^−^ secretion through CFTR, other pH regulatory mechanisms such as HCO_3_^−^/Cl^−^ anion exchanger, electrogenic Na^+^/HCO_3_^−^ cotransporter, and some other electroneutral and/or bicarbonate-independent mechanisms are involved in the IND-induced pH normalization (see Fig. 6).

Generally, the level of CFTR expression at the plasma membrane results from a balance between membrane trafficking, endocytosis, and recycling [12]. Since we did not reveal which step was affected by LPS, further experiments will be necessary. We demonstrated that CFTR inhibitors, NPPB and CFTR_inh172_, abolished the IND-mediated improvement of the ACh-induced or ACh/LPS-induced acidification in pH (Fig. 4c, d). These findings suggest that LPS directly suppressed the cAMP-dependent HCO_3_^−^ secretion through CFTR resulting in acidification of the pH and that the IND-dependent strong activation of CFTR is likely to cause a significant improvement of the LPS-induced acidification in pH. As for other mechanisms, there is a possibility that a CFTR-dependent channel/transporter other than CFTR itself is induced to compensate for the protective effect of IND on CFTR-dependent pH and fluid secretion changes despite the LPS-induced decreases in CFTR expression. Further investigations will be necessary in the future to clarify this issue.

Several studies have provided some indirect evidence for an acquired CFTR dysfunction. *Pseudomonas aeruginosa*, which contains abundant LPS in their cell walls, reduced CFTR-mediated transepithelial anion secretion across polarized human airway epithelial cells by inhibiting the endocytic recycling of CFTR [43]. Various products secreted by *P. aeruginosa*, such as *P. aeruginosa* diffusible material (PsaDM) [48], Pyocyanin [22], PA2394 protein (called CFTR inhibitory factor, Cif) [24], and a *P. aeruginosa* type II secretion system metalloprotease (LasB) [35], reduced the CFTR-dependent currents as well as the plasma membrane expression of CFTR protein in primary human airway epithelial cells. Additionally, cigarette smoke exposure decreased CFTR expression at the gene, protein, and functional levels in Calu-3 cells [8, 41]. Another study has shown that an acquired CFTR dysfunction was present in the lower airways of smokers with and without COPD [11]. Further studies are necessary in order to confirm a causal association between LPS and an acquired dysfunction of CFTR.

Abnormal acidification in the ASL pH is related to a defect in cAMP-dependent HCO_3_^−^ secretion through CFTR [16, 39, 40], and the intracellular regulation of HCO_3_^−^ secretion through CFTR has been well investigated in Calu-3 cells. Bridges and colleagues demonstrated that Calu-3 cells secrete HCO_3_^−^ in response to a cAMP-mediated agonist but can be stimulated to secrete Cl^−^ with a basolateral membrane K^+^ channel-activating agonist such as 1-EBIO (1-ethyl-2-benzimidazolinone), an activator of the basolateral membrane, Ca^2+^-activated charybdotoxin-sensitive, K^+^ channels [5, 10, 18, 45]. Switching between these two secreted anions is determined by the activity of the basolateral K^+^ channel, which can be activated by ACh, as well. If this “switching” mechanism is conserved in the regulation of tracheal gland secretion, it is then likely to explain our findings. When cells are stimulated by ACh or ACh + LPS, tracheal glands secrete HCO_3_^−^ free, Cl^−^ rich, and large amount of fluid, which tend to be relatively acidic in pH. The addition of IND or other LABAs is likely to induce the “switching” mechanism from Cl^−^ to HCO_3_^−^ secretion through CFTR, accelerate singular HCO_3_^−^ secretion, and induce remarkable alkaline changes in pH, although it will in turn simultaneously cause a significant decrease in the amount of fluid (see Fig. 6). If this is true, it would be reasonable that an anti-cholinergic agent such as GLY could further accelerate the IND-mediated improvement of LPS-induced acidification in pH (Fig. 4b).

Our experiments have some advantages in revealing the physiological regulation of airway secretion in vivo. First, we used fresh swine trachea and evaluated their physiological secretory responses induced by as low as 30–100 nM of ACh, which seemed to reproduce the physiological release from vagal nerve endings in the airways in vivo [19, 46]. Second, our findings are based on the effects of clinically used LABAs at appropriate concentrations [9]. Third, we demonstrated changes in the gland secretion rate and the pH using freshly isolated swine tracheal membrane. We assume that these phenomena are likely to occur in vivo, especially in the airways of COPD patients.

There are several limitations in this study. First, we could not perform a comparison between LABAs and LAMAs on the degree of alkaline changes in the ASL pH because the secretions from ACh-stimulated tracheal SMGs were completely suppressed by the addition of LAMA. If LABAs or LAMAs were applied only to the apical side, not to the ACh-containing basolateral side, such comparisons could be performed in the future. Second, the precise mechanisms of LPS on the downregulation of CFTR expression at the plasma membrane were not investigated. There are complicated steps in protein trafficking in polarized epithelia. Third, this study did not investigate whether these bronchodilators really improve the airway acidification and airway defense abnormalities in COPD patients. If the activation of cAMP-dependent HCO_3_^−^ secretion through CFTR plays an important role in maintaining airway secretion suitable for maintenance of the airway defense, it could be a novel therapeutic target for preventing exacerbations of COPD. Fourth, we did not assess the effects of LABA/LAMA other than IND/GLY to confirm the CFTR dependency of LABA/LAMA-induced changes in fluid secretion/pH, because of the difficulties of obtaining other combinations of LABA/LAMA. However, this could become possible in the future.

In conclusion, the present study revealed the adverse effects of LPS on both the quantity and quality of the physiological gland secretion, and the ameliorating effects of LABAs on the LPS-evoked abnormal changes in those properties. Through the activation of CFTR, IND and probably other LABAs play important roles in the maintenance of airway defense against exacerbating factors including LPS. The improvement of an acquired CFTR dysfunction in COPD airways may offer a new therapeutic candidate for reducing the frequency of COPD exacerbations.

## Supplementary Information

Below is the link to the electronic supplementary material.

Supplementary file1 (DOCX 27.2 KB)
