# Supplementary material for: Effects of indacaterol on the LPS-evoked changes in fluid secretion rate and pH in swine tracheal membrane
Source: Pflugers Arch. 2021 May 24;473(6):883–96. doi: 10.1007/s00424-021-02560-z (PMC8164627; doi:10.1007/s00424-021-02560-z)
Supplement: Supplementary file 1 — Supplementary file1 (DOCX 27.2 KB) [file 424_2021_2560_MOESM1_ESM.docx]

**Online Data Supplement**

**Effects of indacaterol on LPS-evoked changes in fluid secretion rate and pH in swine tracheal membrane**

Hidemi Aritake, Tsutomu Tamada, Koji Murakami, Shunichi Gamo, Masayuki Nara, Itsuro Kazama, Masakazu Ichinose and Hisatoshi Sugiura

**Materials and Methods**

**Preparation of swine tracheal tissues**

We used crossbred pigs, LYD (Landrace, Yorkshire and Duroc), aged from 6 to 8 months. The proportions of female and male swine used in the present study were almost even. Details are described in our previous reports [2,7,8,12,13]. Fresh swine tracheas were obtained at a local slaughterhouse and were transported to our laboratory in an ice-cold extracellular solution. The external surfaces of the cartilage portion were cleaned of fat and connective tissues and cut into rings 3-4 cm long. After the cartilage and smooth muscle layers were carefully removed, tracheal tissues with bared SMGs were fixed by pins with the apical wall side up. The apical surface was dried with a gentle air stream from hand dryer, after which 60-80 µl of mineral oil were placed on the surface. The basolateral surfaces were continuously perfused with a warm (37°C) gassed (95%/5%, O_2_/CO_2_) Krebs-Ringer Bicarbonate buffer (KRB). The composition of this solution was (in mM), 120 NaCl, 25 NaHCO_3_, 3.33 KH_2_PO_4_, 0.83 K_2_HPO_4_, 1.2 MgCl_2_, 1.2 CaCl_2_, and 10 glucose (pH 7.4).

**Analysis of the amount of gland secretion in swine tracheal membrane**

Using an optical method reported by Joo NS and colleagues [6], we succeeded in the real-time evaluation of the amount of physiologic airway secretion from swine tracheal SMGs. Under a stereoscopic microscope, ACh-induced airway secretion from individual SMGs was observed as hillock formations on a mineral oil layer coating the airway surface. We measured the total amount of the hillock size per unit area (25mm^2^) and expressed them in nl/25mm^2^ (Figure 1d-e). The effects of several drugs were compared by estimating the mean values of the ACh (100 nM) responses at 180 sec as 100% to exclude artifacts from differences in the degree of secretory responses of individual swine trachea (Figure 2a, 2c).

**Preparation of cells**

For patch-clamp experiments, fresh SMG cells were isolated from swine tracheas and dispersed enzymatically into single or clustered acinar cells. Details are described in our previous reports [2,5,7,8,12,13]. After dispersion and washing with centrifugation at 180 *g*, the cells were resuspended in a standard extracellular solution (in mM) of 120 NaCl, 4.7 KCl, 1.13 MgCl_2_, 1.2 CaCl_2_, 10 glucose, and 10 HEPES until use. Calu-3 cells were purchased from American Type Culture Collection (ATCC, Rockville, MD) and grown in Dulbecco’s modified Eagle’s medium and Ham’s F-12 (1:1) supplemented with 15% fetal bovine serum and 2 mM glutamine. The cells were incubated in a humidified atmosphere containing 5% CO_2_ at 37 °C. Calu-3 cells were grown on Snapwell filters as previously described [11] and studied in a horizontal chamber.

**Electrophysiology**

Ionic currents were measured according to a standard whole-cell mode patch-clamp technique using a patch-clamp amplifier (EPC9; HEKA Electronic, Lambrecht/Pfalz, Germany), low-pass filtered at 2.9 kHz, and monitored on both a built-in software oscilloscope and a pen recorder (Electronic Recorder R-03Ka; Rikadenki, Tokyo, Japan). Details are described in our previous reports [2,5,7,8,12,13]. Membrane currents were monitored at two different holding potentials (Hps), that is, at 0 and -80 mV, which roughly corresponded to the chloride (Cl^-^) and potassium (K^+^) equilibrium potential, respectively, under the present electrolyte conditions. This was accomplished by applying 200-ms voltage pulses of -80 mV at a frequency of 2 Hz to the pipette HP of 0 mV (Pulsefit version.8.11, HEKA). The upward deflection of the current tracing corresponds to Ca^2+^-dependent outward K^+^ flux and the downward deflection corresponds to Ca^2+^-dependent outward Cl^-^ flux. These currents are called the outward current (*I_o_*) and inward current (*I_i_*), respectively. Using proper channel inhibitors and ion substitution experiments, we have reported that the ACh-induced *I_o_* and *I_i_* were carried mainly by K^+^ and Cl^-^, respectively, which were dependent on [Ca^2+^]_i_ [5,9,10]. The composition of the extracellular (bath) solution was described above, and that of the intracellular (pipette) solution was (in mM), 120 KCl, 1.13 MgCl_2_, 0.5 EGTA, 1Na_2_ATP, 10 glucose and 10 HEPES. The fluids were superfused over the cells by hydrostatic pressure-driven application (20–30 cmH_2_O) through polyethylene tubes. These solutions were at pH 7.2 and all experiments were carried out at room temperature.

**Quantification procedure**

The procedure to evaluate the ionic responses was also applied in our previous reports [2,5,7,8,12,13]. Briefly, we first measured the area circumscribed with the current trace (*I_o_* or *I_i_*) and baseline for 20 s (= Area Under Curve_20_) using a digital planimeter (PLACOM KP-92N; Koizumi, Tokyo, Japan). This area shows the net electric charge movements of 20 s duration in each condition and the effects of IND on the ACh-stimulated responses were estimated by comparing just before and after treatment with IND and was expressed as a percentage of the pretreatment control values.

**Analysis of the ASL pH in swine tracheal membrane**

Individual SMG secretions were collected from swine tracheal membrane overlaid with a mineral oil. A mixture of collected airway secretions and pH indicator, SNARF-1 (Thermo Fisher Scientific, Waltham, MA, USA) [3], was covered with oil in micropipettes and isolated from air until use so that the pH values remained unchanged throughout the experiments. The fluorescence readings (excitation at 488 and emission at 510 and 640 nm) were carried out in a Flexstation 3 microplate reader (Molecular Devices, Sunnyvale, CA, USA), every 30 sec for 360 sec, and converted to pH values based on the pH reference solutions.

**Analysis of apical surface liquid pH on Calu-3 cells**

Calu-3 cells with double barrel voltage and pH electrodes were used in a horizontal chamber that allowed for estimate of the pH values just above the apical membrane of the Calu-3 cells. Details are described in previous reports [1,4,11]. The microelectrodes were pulled from quartz theta glass capillaries (QT120-90-7.5, Sutter Instrument, Novato, CA, USA) with a vertical laser-based micropipette puller (P-2000, Sutter Instrument). The pH-selective barrel was selectively silanized with hexamethyldisilazane (Sigma-Aldrich, St Louis, MO, USA). Then, a tiny drop of hydrogen ionophore I-cocktail B (Fluka 95293; Sigma-Aldrich) was put into the tip of pH-selective barrel. The voltage and pH-selective barrels were backfilled with an electrolyte solution of 150 mM KCl. The pH values were calculated from the potential differences between two barrels. The pH electrode was calibrated before and after the experiment in a series of standard solutions (pH=7.5, 7.0 and 6.5) and confirmed to have a good slope response of −61.6 mV per unit pH change. All recordings were carried out with a high input impedance electrometer, FD 223 Electrometer (World Precision Instruments, Sarasota, FL, USA). The double barrel voltage and pH electrodes were moved vertically using a micromanipulator (MP 285, Sutter Instrument) and mounted carefully at 25 μm above the apical surface of the Calu-3 cells. The buffer capacity of the apical solution was adjusted to become low enough to detect very small changes in the pH. The composition of the apical solution with low buffering capacity was (in mM), 145 NaCl, 1.6 KH_2_PO_4_, 0.4 K_2_HPO_4_, 1.2 MgCl_2_, 1.2 CaCl_2_, and 10 mannitol (pH 7.4). The basolateral surfaces were continuously perfused with gassed (95%/5%, O_2_/CO_2_) KRB buffer and the composition was (in mM), 120 NaCl, 25 NaHCO_3_, 3.33 KH_2_PO_4_, 0.83 K_2_HPO_4_, 1.2 MgCl_2_, 1.2 CaCl_2_, and 10 mannitol (pH 7.4). All solutions were warmed at 37°C.

**Immunofluorescence staining**

Immunofluorescence staining was performed to detect CFTR as described previously [2,7,8]. Briefly, tissue blocks of swine tracheas were fixed with 10% paraformaldehyde in PBS for 24 hours at room temperature after they were incubated in a medium containing LPS (100 μg/ml) with or without IND (1 µM) for 10 min. Tissue sections were deparaffinized through graded alcohols and washed in PBS. Specimens were permeabilized with a heat-induced antigen retrieval method using Tris EDTA buffer, an ethylenediaminetetraacetic acid (EDTA) solution buffered at pH 9 (10 mM Tris Base, 1 mM EDTA solution, 0.05% Tween 20, and NaOH for titration to pH 9), at 121 °C for 15 min in an autoclave. For fluorescence labeling of the cell membrane, specimens were incubated with 5 μg/ml wheat germ agglutinin (WGA) 594 conjugate (1:1200 dilution, Invitrogen, Waltham, MA, USA) for 10 min at room temperature. After blocking with 1 % skim milk in 10 mM PBS for 30 min at room temperature, the specimens were incubated with mouse monoclonal anti-CFTR antibody (CF3, 1:500 dilution, Abcam, Cambridge, UK) overnight at 4°C. After being washed, the specimens were incubated with donkey anti-mouse IgG with FITC (1:200 dilution, Abcam) for 30 min at room temperature. The Hoechst (Thermo Fisher Scientific) reaction was used to visualize the immune positive cells. Specimens were mounted by VECTASHILD Antifade Mounting Medium (Vector Laboratories Inc. Burlingame, CA, USA). Slides were evaluated using a multiphoton confocal LSM780 NLO microscope system (Carl Zeiss, Jena, Germany) and photographed using a digital camera.

**Western blotting**

Western blotting was performed to detect CFTR as described previously [2,7,8]. Calu-3 cells grown on 6-well culture plates were incubated in a medium containing LPS (0, 10 and 100 μg/ml) with or without IND (1 µM) for 3 hrs. Cells were lysed in RIPA buffer containing protease inhibitor cocktail (Sigma-Aldrich) and centrifuged at 13,000 rpm for 10 min at 4 °C. 20 μg proteins per well were loaded and separated by SDS-PAGE (7.5% polyacrylamide gel; Bio-Rad, Herclues, CA, USA) and transferred onto polyvinylidene fluoride membranes (Millipore, Darmstadt, Germany). The membranes were then blocked with a Blocking Reagent (Toyobo, Osaka, Japan) and then immunoblotted with mouse monoclonal anti-CFTR antibody (M3A7; 1:500 dilution, Abcam, Cambridge, UK) or anti-β-actin antibody (1:5000 dilution; Upstate Biotechnology, Lake Placed NY, USA) at 4 °C overnight followed by the relevant horse radish peroxidase-conjugated secondary antibodies (1:2000 dilution, Santa Cruz Biotechnology, Dallas, TX, USA) for 1 h at room temperature. The signals were visualized using the ECL plus Western Blotting Reagent (Amersham Biosciences, Buckinghamshire, UK) and detected with a luminescent image analyzer (LAS-4000; Fujifilm, Tokyo, Japan). Band intensity was quantified by ImageJ 1.52v software (National Institutes of Health, Bethesda, MD, USA).

**Statistical analysis**

All analyses were performed by means of JMP Pro 14 (SAS Institute Inc., Cary, NC, USA). The data are expressed as means ± standard errors (SE); n is the number of experiments in different animals. Electrophysiological experiments were analyzed by the Wilcoxon signed rank test. The amount of airway secretion and the ASL pH were analyzed by the Wilcoxon signed rank test and matched paired t test, respectively. Statistical significance was accepted at *p* < 0.05, indicated by asterisks or other symbols in all figures.

**Reagents**

Indacaterol was provided by Novartis Pharma AG (Basel, Switzerland). HEPES was purchased from Dojindo (Kumamoto, Japan). Collagenase was from Wako Pure Chemicals (Osaka, Japan). 5-nitro-2-(3-phenylpropyl-amino) benzoate (NPPB) and 3-[(3-trifluoromethyl)phenyl]-5-[(4-carboxyphenyl)methylene]-2-thioxo-4-thiazolidinone (CFTR_inh172_) were from R&D Systems (Minneapolis, USA). All other chemicals were purchased from Sigma Aldrich.

**REFERENCES**

1. Bridges RJ (2012) Mechanisms of Bicarbonate Secretion: Lessons from the Airways. Csh Perspect Med 2. doi:ARTN a015016.10.1101/cshperspect.a0150161.

2. Gamo S, Tamada T, Murakami K, Muramatsu S, Aritake H, Nara M, Kazama I, Okazaki T, Sugiura H, Ichinose M (2018) TLR7 agonist attenuates acetylcholine-induced, Ca^2+^ -dependent ionic currents in swine tracheal submucosal gland cells. Exp Physiol 103:1543-1559. doi:10.1113/ep087221

3. Han J, Loudet A, Barhoumi R, Burghardt RC, Burgess K (2009) A ratiometric pH reporter for imaging protein-dye conjugates in living cells. J Am Chem Soc 131:1642-1643. doi:10.1021/ja8073374

4. Hug MJ, Tamada T, Bridges RJ (2003) CFTR and bicarbonate secretion to epithelial cells. News Physiol Sci 18:38-42

5. Iwase N, Sasaki T, Oshiro T, Tamada T, Nara M, Sasamori K, Hattori T, Shirato K, Maruyama Y (2002) Differential effect of epidermal growth factor on serous and mucous cells in porcine airway submucosal gland. Resp Physiol Neurobiol 132:307-319. doi: 10.1016/s1569-9048(02)00118-0

6. Joo NS, Wu JV, Krouse ME, Saenz Y, Wine JJ (2001) Optical method for quantifying rates of mucus secretion from single submucosal glands. Am J Physiol Lung Cell Mol Physiol 281:L458-468. doi:10.1152/ajplung.2001.281.2.L458

7. Murakami K, Tamada T, Nara M, Muramatsu S, Kikuchi T, Kanehira M, Maruyama Y, Ebina M, Nukiwa T. Toll-like receptor 4 potentiates Ca^2+^-dependent secretion of electrolytes from swine tracheal glands. *Am J Respir Cell Mol Biol* 2011;45:1101-1110. doi. org/ 10. 1165/ rcmb. 2011- 0020OC

8. Muramatsu S, Tamada T, Nara M, Murakami K, Kikuchi T, Kanehira M, Maruyama Y, Ebina M, Nukiwa T, Ichinose M. Flagellin/TLR5 signaling potentiates airway serous secretion from swine tracheal submucosal glands. *Am J Physiol Lung Cell Mol Physiol* 2013;305:L819-L830. doi. org/ 10. 1152/ ajplu ng. 00053.2013

9. Sasaki T, Shimura S, Wakui M, Ohkawara Y, Takishima T, Mikoshiba K (1994) Apically localized IP_3_ receptors control chloride current in airway gland acinar cells. Am J Physiol 267:L152-158. doi: 10.1152/ajplung.1994.267.2.L152.

10. Sasamori K, Sasaki T, Takasawa S, Tamada T, Nara M, Irokawa T, Shimura S, Shirato K, Hattori T (2004) Cyclic ADP-ribose, a putative Ca^2+^-mobilizing second messenger, operates in submucosal gland acinar cells. Am J Physiol Lung Cell Mol Physiol 287:L69-78. doi:10.1152/ajplung.00454.2003

11. Tamada T, Hug MJ, Frizzell RA, Bridges RJ (2001) Microelectrode and impedance analysis of anion secretion in Calu-3 cells. JOP 2:219-228

12. Tamada T, Nara M, Kanatsuka H, Nagaoka M, Koshida R, Tamura G, Hattori T (2007) A potentiating effect of endogenous NO in the physiologic secretion from airway submucosal glands. Am J Respir Cell Mol Biol 37:357-365. doi:10.1165/rcmb.2006-0389OC

13. Tamada T, Sasaki T, Saitoh H, Ohkawara Y, Irokawa T, Sasamori K, Oshiro T, Tamura G, Shimura S, Shirato K (2000) A novel function of thyrotropin as a potentiator of electrolyte secretion from the tracheal gland. Am J Resp Cell Mol Biol 22:566-573. doi: 10.1165/ajrcmb.22.5.3847
